# The G Protein Coupled Receptor 3 Is Involved in cAMP and cGMP Signaling and Maintenance of Meiotic Arrest in Porcine Oocytes

**DOI:** 10.1371/journal.pone.0038807

**Published:** 2012-06-07

**Authors:** Cai-Rong Yang, Yanchang Wei, Shu-Tao Qi, Lei Chen, Qing-Hua Zhang, Jun-Yu Ma, Yi-Bo Luo, Ya-Peng Wang, Yi Hou, Heide Schatten, Zhong-Hua Liu, Qing-Yuan Sun

**Affiliations:** 1 College of Life Science, Northeast Agricultural University of China, Harbin, China; 2 State Key Laboratory of Reproductive Biology, Institute of Zoology, Chinese Academy of Sciences, Beijing, China; 3 Department of Veterinary Pathobiology, University of Missouri, Columbia, Missouri, United States of America; University of Connecticut, United States of America

## Abstract

The arrest of meiotic prophase in mammalian oocytes within fully grown follicles is dependent on cyclic adenosine monophosphate (cAMP) regulation. A large part of cAMP is produced by the Gs-linked G-protein-coupled receptor (GPR) pathway. In the present study, we examined whether GPR3 is involved in the maintenance of meiotic arrest in porcine oocytes. Expression and distribution of GPR3 were examined by western blot and immunofluorescence microscopy, respectively. The results showed that GPR3 was expressed at various stages during porcine oocyte maturation. At the germinal vesicle (GV) stage, GPR3 displayed a maximal expression level, and its expression remained stable from pro-metaphase I (MI) to metaphase II (MII). Immunofluorescence staining showed that GPR3 was mainly distributed at the nuclear envelope during the GV stage and localized to the plasma membrane at pro-MI, MI and MII stages. RNA interference (RNAi) was used to knock down the GPR3 expression within oocytes. Injection of small interfering double-stranded RNA (siRNA) targeting GPR3 stimulated meiotic resumption of oocytes. On the other hand, overexpression of GPR3 inhibited meiotic maturation of porcine oocytes, which was caused by increase of cGMP and cAMP levels and inhibition of cyclin B accumulation. Furthermore, incubation of porcine oocytes with the GPR3 ligand sphingosylphosphorylcholine (SPC) inhibited oocyte maturation. We propose that GPR3 is required for maintenance of meiotic arrest in porcine oocytes through pathways involved in the regulation of cAMP and cGMP.

## Introduction

Mammalian oocyte entry into meiosis occurs early in oogenesis, but arrests in the first meiotic prophase until fully competent to resume meiosis [1], [2]. Meiotic resumption needs luteinizing hormone (LH) from the pituitary to act on the somatic cells of the follicle surrounding the oocytes [3], [4], [5]. Oocytes arrested at the prophase diplotene stage of meiosis I acquire the ability to resume meiosis as they approach their full size [6]. In response to LH, meiotic resumption occurs: the chromosomes condense, the nuclear envelope breaks down, and a metaphase spindle forms.

Oocytes are maintained at meiotic prophase by inherent factors, which correlate with low levels of cell cycle regulatory protein activity, including cyclin B and CDC2 [5], [7], [8]. It is generally accepted that cAMP is an important mediator of LH action for inducing oocyte meiotic resumption [1], [9], [10]. A high level of cAMP blocks spontaneous meiotic resumption via activating cAMP-dependent protein kinase (PKA) [11], [12], [13]. In porcine oocytes, meiotic arrest was also maintained by culture of cumulus-oocyte complexes (COCs) with drugs for stimulating cAMP production, for example, a phosphodiesterase (PDE) inhibitor (3-isobtyl-1-methylxanthine, IBMX; hypoxanthine, HX), an activator of adenylate cyclase (forskolin), and a cAMP analog (dibutyryl cAMP) [12], [14], [15]. In prophase-arrested oocytes, the activated cAMP-PKA directly targets Cdc25B phosphorylation and results in inhibition of the cyclin B-Cdk1 complex [10], [16], which are components of the maturation promoting factor (MPF) [13], [17]. On the other hand, activation of MPF drives the prophase-to-metaphase transition [7], [18], [19]. Furthermore, cyclic guanosine monophosphate (cGMP) can also affect cAMP concentration through activation and inactivation of PDEs. cGMP stimulates PDE2 activity whereas it inhibits PDE3 activity [20], [21], resulting in increase in cAMP levels and activation of cAMP-dependent signaling [22], which then facilitates the blockage of meiotic resumption.

cAMP produced by the oocyte itself is primarily required for meiotic arrest through the activation of a guanine nucleotide-binding proteins (G proteins) Gs and adenylyl cyclase [3], [23], [24]. Gs by itself has no detectable constitutive activity, but it is stimulated by an orphan G-protein coupled receptor-GPR3 in the oocytes to keep Gs active [7], [8]. Evidence exists that GPR3 is a constitutive activator of adenylyl cyclase in cultured cells [25]. In mouse oocytes, GPR3 can activate Gs and elevate cAMP levels, arresting the progress to meiotic resumption [8]. GPR3 is mainly localized in oocytes, rather than in the follicle cells in the mouse [3], [7], and oocytes from GPR3 knockout mice resume meiosis within antral follicles, independent of an increase in luteinizing hormone, and this phenotype can be reversed by injecting GPR3 mRNA into the oocytes [7]. It was also confirmed that downregulation of GPR3 and GPR12 through morpholino oligonucleotides injection caused meiotic resumption in mouse and rat oocytes [3]. Taken together, the above findings strongly suggest that meiotic arrest is dependent on continuous cAMP signaling through activating GPR3/Gs/adenylyl cyclase endogenous to the oocytes. If any of the components of this signaling pathway is eliminated in the follicle-enclosed oocytes, spontaneous maturation will occur [3], [7], [23], [24], [26]. As a preeminent animal model, study on the relationship between GPR3 and meiotic resumption in porcine oocytes benefits studies on human oocytes because of the many similarities between porcine and human oocytes regarding physiology and immunology, porcine and human has similar in oocyte size, spindle appearance, lipid dropt distribution, and body size. They also have similar sequence integrity in specific part of genome. To the best of our knowledge, no study has yet investigated the role of GPR3 in meiotic progression of porcine oocytes.

Here, we examined the expression and localization of GPR3 during porcine oocyte meiotic maturation. We also showed its critical roles in regulating meiotic prophase arrest by protein depletion and overexpression approaches.

## Results

### Subcellular localization and expression of GPR3 in porcine oocytes during meiotic maturation

**Figure 1:**
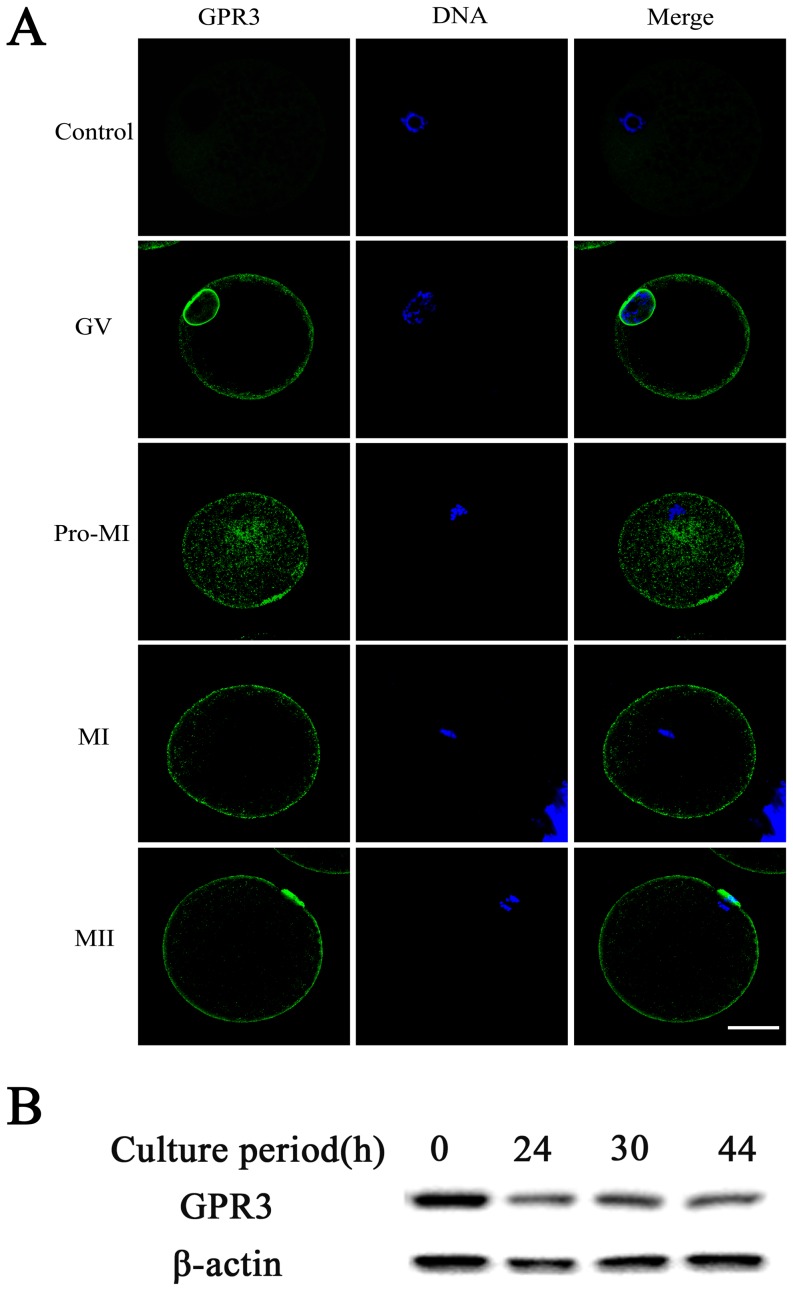
Expression and subcellular localization of GPR3 during porcine oocyte meiotic maturation. (A) Subcellular localization of GPR3 as revealed by immunofluorescent staining. In the GV stage, GPR3 was mainly distributed at the nuclear membrane and plasma membrane. GPR3 accumulated at the inner cytoplasm and plasma membrane at the pro-MI stage. From MI to MII, GPR3 aggregated at the plasma membrane. (B) Expression of GPR3 protein was measured by western blotting. Samples were collected at 0 h, 24 h, 30 h, and 44 h of culture which were the time points when most oocytes had reached the GV, GVBD, MI and MII stages, respectively. Each sample was derived from 150 oocytes. Green, GPR3; Blue, chromatin. Bar = 40 µm.

To determine the function of GPR3 during meiosis, subcellular localization and expression were examined. The localization of GPR3 in porcine oocytes is shown in Fig. 1A. In GV oocytes, the prominent staining of GPR3 was mainly concentrated at the nuclear membrane, and the remaining GPR3 accumulated at the plasma membrane. After germinal vesicle breakdown (GVBD), the positive signal of GPR3 was observed evenly in the inner cytoplasm and plasma membrane. In the MI and MII stages, GPR3 was distributed at the plasma membrane. In control group, we did not detect any green positive signal. For affirming the subcellular localization of GPR3 in GV stage, we scanned the oocytes thoroughly in numerous planes and obtained the same result with the single plane (see Fig. S1). Western blot analysis showed that GPR3 was expressed in all porcine oocyte stages. As shown in Fig. 1B, the GPR3 expression displayed a maximal level at the GV stage, and remained stable from GVBD to the MII stages.

### Overexpression of GPR3 causes meiotic arrest in porcine oocytes

**Figure 2:**
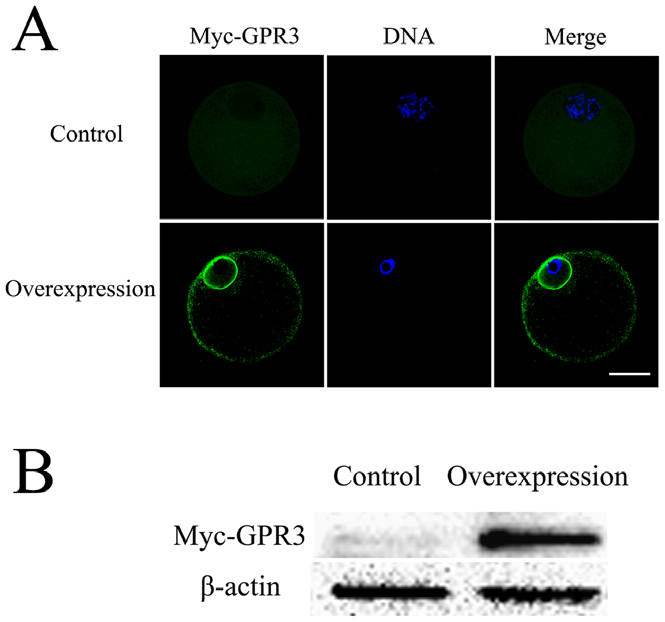
The efficiency of GPR3 overexpression. (A) Immunofluorescence detection of GPR3 overexpression. Control: oocytes were injected with 2.5 mg/ml Myc_6_ mRNA solution; Overexpression: oocyets were injected with 2.5 mg/ml Myc_6_-GPR3 mRNA solution. Green, Myc_6_-GPR3; Blue, chromatin. Bar = 40 µm. (B) Samples from control and overexpression groups were collected to test the expression of Myc_6_-GPR3. Control: 150 oocytes injected with 2.5 mg/ml Myc_6_ mRNA solution; Overexpression: 150 oocytes injected with 2.5 mg/ml Myc_6_- GPR3 mRNA solution. Oocytes were then incubated for 15 h in TCM-199 medium containing 2.5 µM Milrinone before collection or culture for western blotting.

We next examined the effect of GPR3 overexpression on meiotic resumption. Immunofluorescence was used to confirm the overexpression of Myc_6_-GPR3. Oocytes injected with the same amount of Myc_6_ mRNA were used as control, which did not result in a specific signal (Fig. 2A). Successful overexpression was observed in the Myc_6_-GPR3 mRNA injection group (Fig. 2A). Myc_6_-GPR overexpression was also confirmed by western blot. A clear band was detected after Myc_6_-GPR3 mRNA injection, whereas injection of Myc_6_ mRNA in the control group did not result in specific band detection (Fig. 2B).

**Figure 3:**
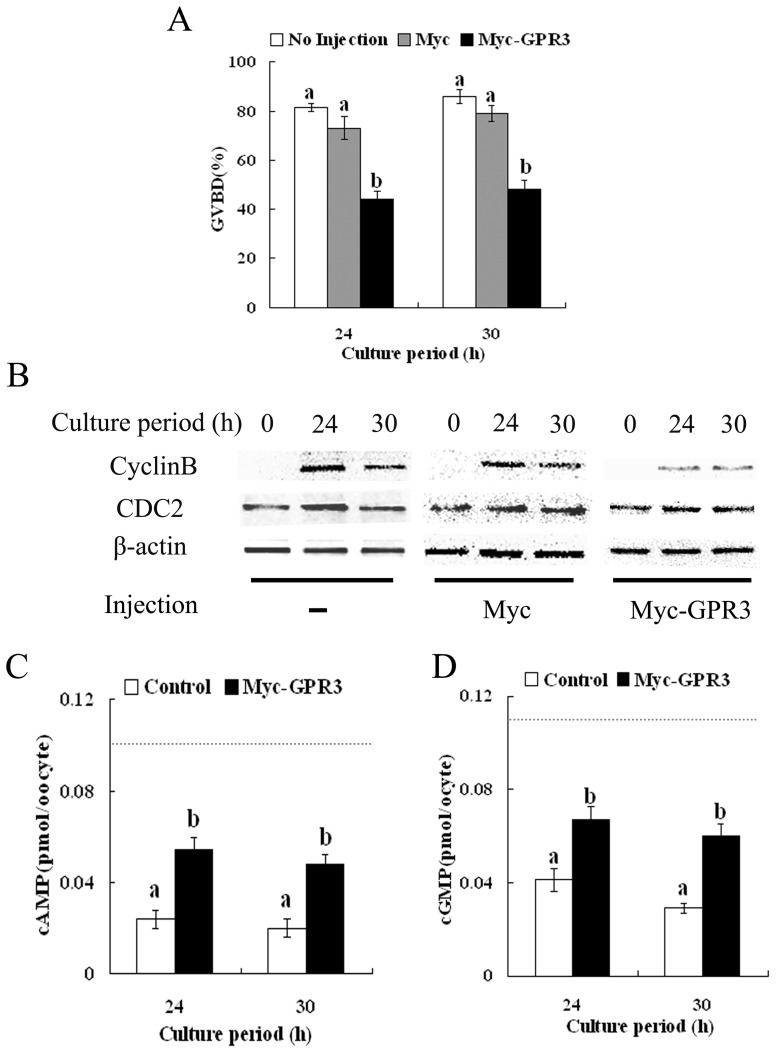
Overexpression of GPR3 inhibits meiotic resumption in porcine oocytes. (A) Oocytes cultured in normal medium without HX were injected with 2.5 mg/ml Myc_6_-GPR3 or control Myc_6_ and then cultured for 24 h or 30 h. The GVBD rates of the injected oocytes are shown along with non-injected oocytes. (B) Cyclin B (upper panel) and CDC2 (middle panel) levels were detected by western blot using 150 oocytes in each sample. The results are shown along with those of non-injected oocytes cultured without HX. Samples were collected after culture in medium without HX at 0 h, 24 h and 30 h. (C) cAMP levels of porcine oocytes in the Myc_6_-GPR3 injection group and control Myc_6_ injection group after culture without HX at 24 h or 30 h. Dotted line represents cAMP level at time 0. (D) cGMP levels of porcine oocytes in the Myc_6_-GPR3 injection group and control Myc_6_ injection group after culture without HX at 24 h or 30 h. Dotted line represents cGMP level at time 0. The number “n” on top of the bars indicates the total number of treated oocytes in each group. Data are shown as mean ± SEM of at least three repeated experiments and letters ‘a’ and ‘b’ indicate statistically significant difference (p<0.05).

To further study the effect of GPR3 overexpression on arrest of meiotic resumption, we examined the GVBD rate after overexpression. In our culture system without HX, 81.42% of the oocytes underwent GVBD at 24 h of culture and the rate reached 85.87% at 30 h (Fig. 3A). Injection of Myc_6_ mRNA did not significantly change these rates (72.95% and 78.95%, respectively). However, when oocytes were injected with Myc_6_-GPR3 mRNA, the GVBD rates were significantly decreased to 44.34% and 48.37% at 24 h and 30 h, respectively. These results suggested that overexpression of GPR3 inhibited meiotic resumption.

### Overexpression of GPR3 decreases cyclin B level in porcine oocytes

Cyclin B and CDC2 expression was further analyzed in GPR3 overexpression oocytes. As presented in Fig. 3B, western blot analysis showed that cyclin B was undetected in GV oocytes at 0 h; however, it increased dramatically in culture medium without HX at 24 h and 30 h, respectively, while the CDC2 level remained almost unchanged. Injection of Myc_6_ mRNA did not significantly change the expression pattern of both proteins either. However, compared to control groups, cyclin B level in Myc_6_-GPR3 mRNA-injected oocytes was evidently decreased at 24 h and 30 h of culture, while the CDC2 level was apparently unchanged. These results were consistent with the inhibitory effect of GPR3 overexpression on meiotic resumption.

### Overexpression of GPR3 increases cAMP levels and cGMP levels in porcine oocytes

To investigate whether GPR3 overexpression was involved in cAMP signaling and thus affected meiotic resumption, we determined cAMP levels and cGMP levels after Myc_6_-GPR3 mRNA injection. cAMP level was 0.098 pmol/oocyte in average at 0 h. As shown in Fig. 3C, compared with Myc_6_ mRNA injection, the cAMP level was significantly increased after GPR3 overexpression. When oocytes were injected with Myc_6_ mRNA, the concentration of cAMP was 0.024 pmol/oocyte and 0.02 pmol/oocyte in average at 24 h and 30 h of culture without HX, whereas after Myc_6_-GPR3 mRNA injection, cAMP level increased to 0.055 pmol/oocyte and 0.048 pmol/oocyte in average at 24 h and 30 h, respectively.

The tendency of the cGMP level was similar to the cAMP level. cGMP level was 0.116 pmol/oocyte in average at 0 h. As presented in Fig. 3D, the concentration of cGMP was 0.041 pmol/oocyte and 0.029 pmol/oocyte in average at 24 h and 30 h of culture without HX after Myc_6_ mRNA injection, however, when oocytes were injected with Myc_6_-GPR3 mRNA, the quantity significantly increased to 0.067 pmol/oocyte and 0.06 pmol/oocyte, respectively.

### Depletion of GPR3 by RNAi causes meiotic resumption

**Figure 4:**
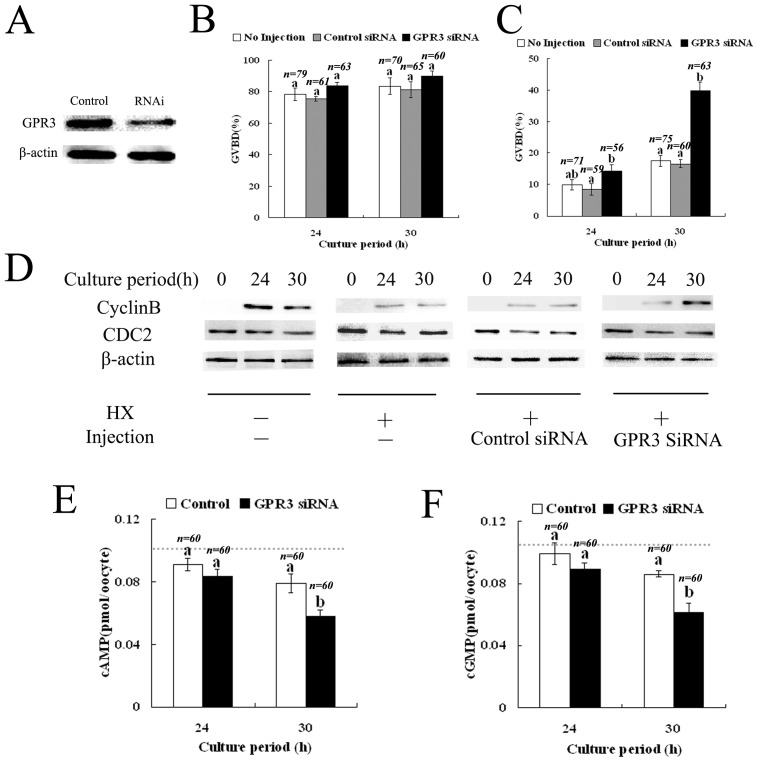
The effect of GPR3 RNAi on meiotic resumption in porcine oocytes. (A) Samples from control and RNAi groups were collected to test the efficiency of GPR3-RNAi. Control: 150 oocytes injected with 25 µM control siRNA; RNAi: 150 oocytes injected with 25 µM GPR3 siRNA. Oocytes were then incubated for 24 h or 30 h in the TCM-199 medium with or without 4 mM HX before collection for western blotting. (B) Oocytes cultured with normal medium without HX were injected with 25 µM GPR3 siRNA or control siRNA and then cultured for 24 h or 30 h. The GVBD rates of the injected oocytes are shown along with non-injected oocytes. (C) Oocytes treated with 4 mM HX were injected with the GPR3 siRNA or control siRNA and then cultured in the presence of HX for 24 h or 30 h. The GVBD rates of the injected oocytes are shown along with non-injected oocytes. (D) Cyclin B (upper panel) and CDC2 (middle panel) levels were detected after siRNA injection by western blot using 150 oocytes in each sample. The results are shown along with those of non-injected oocytes cultured without HX. Samples were collected from culture medium with or without HX at 0 h, 24 h and 30 h. (E) cAMP levels of porcine oocytes in the GPR3 siRNA injection group and control siRNA group after culture with HX at 24 h or 30 h. Dotted line represents cAMP level at time 0. (F) cGMP levels of porcine oocytes in the GPR3 siRNA injection group and control siRNA group after culture with HX at 24 h or 30 h. Dotted line represents cGMP level at time 0. The number “n” on top of the bars indicates the total number of treated oocytes in each group. Data are shown as mean ± SEM of at least three repeated experiments and letters ‘a’ and ‘b’ indicate statistically significant difference (p<0.05).

We further used RNAi to investigate the effects of GPR3 downregulation on meiosis progression. The GV stage oocytes were maintained in meiotic arrest by incubation in medium containing HX; they were injected with GPR3 siRNA or negative control siRNA, and the meiotic progression was followed up to 48 h. RNAi efficiency was detected by western blot. Compared with the control groups (control siRNA injection), the GPR3 expression in siRNA-injected oocytes was decreased to 27% (Fig. 4A), revealing successful downregulation.

In our culture system without HX, 78.32% of intact oocytes underwent GVBD at 24 h and the rate reached 83.52% at 30 h of culture. Injection of control siRNA did not significantly change these rates (75.48% and 81.22%, respectively). As presented in Fig. 4B, the GVBD rates of the GPR3 siRNA injected oocytes were 83.89% at 24 h and 89.82% at 30 h, respectively.

When porcine oocytes were cultured in inhibition-induction maturation medium with HX (Fig. 4C), only 9.94% and 17.48% of non-injected oocytes underwent GVBD at 24 h and 30 h, respectively. Injection of control siRNA did not affect both rates (8.55% and 16.61%, respectively). As shown in Fig. 4C, HX indeed delayed meiotic resumption of porcine oocytes. However, injection of GPR3 siRNA significantly accelerated meiotic resumption of the porcine oocytes, and the GVBD rates recovered to 14.39% at 24 h and 39.82% at 30 h of culture.

### Depletion of GPR3 by siRNA injection increases cyclin B level in porcine oocytes

To further examine the detailed correlation between GPR3 and meiotic resumption, we analyzed cyclin B and CDC2 levels in porcine oocytes with GPR3 siRNA injection. As shown in Fig. 4D, cyclin B was undetected in porcine oocytes at the GV stage, but dramatically increased after culture for 24 h and 30 h in normal maturation medium, while the CDC2 level remained almost unchanged. When control siRNA-injected porcine oocytes were cultured in medium containing HX (Fig. 4D), only faint bands of cyclin B were detected until 24 h and 30 h of culture. These results were consistent with the inhibitory effect of HX on meiotic resumption. However, in spite of the presence of HX, cyclin B bands were clearly observed in the porcine oocytes injected with GPR3 siRNA at 24 h and 30 h of culture (Fig. 4D), respectively. The CDC2 level of oocytes with GPR3 siRNA-injection remained relatively stable.

### Depletion of GPR3 by siRNA injection decreases cAMP levels and cGMP levels in porcine oocytes

Downregulation of GPR3 by RNAi significantly decreased cAMP levels (Fig. 4E). cAMP level was 0.104 pmol/oocyte in average at 0 h. The concentrations of cAMP in control siRNA-injected oocytes were 0.091 pmol/oocyte and 0.079 pmol/oocyte at 24 h and 30 h of culture with HX, respectively. However, the cAMP levels decreased to 0.083 pmol/oocyte and 0.058 pmol/oocyte at the same time points after injection with GPR3 siRNA.

We also determined the cGMP levels after siRNA-injection. cGMP level was 0.112 pmol/oocyte in average at 0 h. As shown in Fig. 4F, in oocytes injected with control siRNA, the concentrations of cGMP were 0.099 pmol/oocyte and 0.086 pmol/oocyte, respectively, at 24 h and 30 h of culture with HX. However, cGMP levels decreased to 0.089 pmol/oocyte and 0.061 pmol/oocyte at the same time points after injection with GPR3 siRNA.

### The localization of GPR3 is not affected by ligand

**Figure 5:**
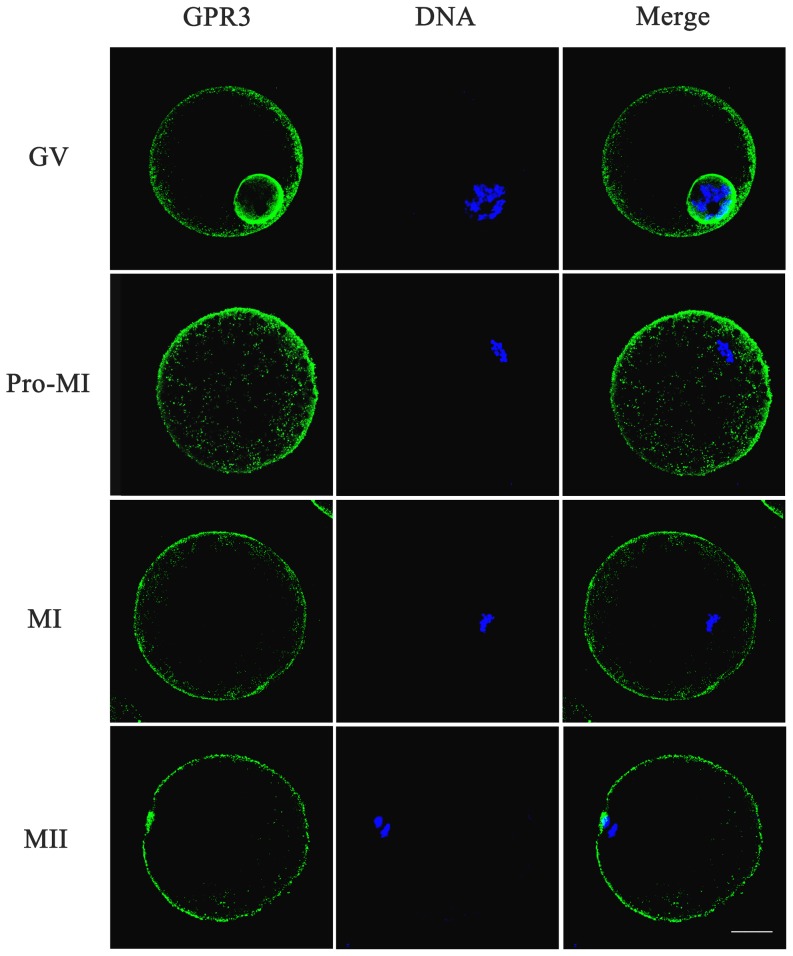
The effect of SPC on distribution of GPR3 in porcine oocytes. Distribution of GPR3 in oocytes after treatment with SPC was revealed by immunofluorescent staining. In the GV stage, GPR3 was mainly distributed at the nuclear membrane and plasma membrane. GPR3 accumulated in the inner cytoplasm and plasma at the pro-MI stage. From MI to MII stages, GPR3 aggregated at the plasma membrane. Green, GPR3; Blue, chromatin. Bar = 40 µm.

To detect whether a GPR3 ligand, sphingosylphosphorylcholine (SPC), affects the localization of GPR3, we used immunofluorescence staining to investigate the distribution of GPR3 after ligand treatment. As shown in Fig. 5, GPR3 mainly distributed at nuclear membrane at the GV stage, while some GPR3 also distributed in the plasma membrane. At pro-MI stage, the positive signal of GPR3 was detected in the inner cytoplasm and plasma membrane. In the MI and MII stages, GPR3 was distributed at the plasma membrane. The distribution pattern of GPR3 was the same as that of oocyte under normal culture, indicating that ligand treatment did not affect the localization of GPR3.

### GVBD is delayed by a ligand for GPR3

**Figure 6:**
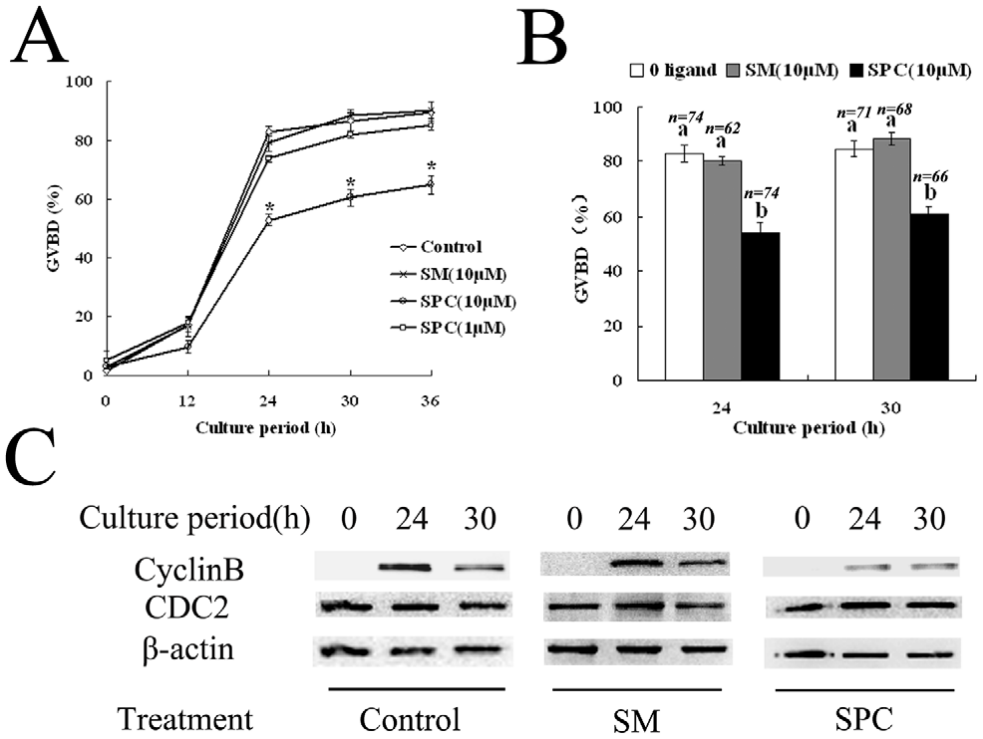
The effect of SPC and SM on meiotic resumption in porcine oocytes. (A) Porcine oocytes were collected in TCM-199 medium with or without 1 or 10 µM SPC or 10 µM SM and then placed in 200 µl drops under mineral oil. Oocytes were scored for meiotic maturation at 0 h, 12 h, 24 h, 30 h, and 36 h. (B) GVBD rates of porcine oocytes treated with 10 µM SPC, 10 µM SM or without ligand 24 h or 30 h of isolation. The number “n” on top of the bars indicates the total treated oocytes in each group. Letters ‘a’, ‘b’ and the symbol “*” indicate statistically significant difference (P<0.05). Data represent the mean ± SEM of at least three repeated experiments. (C) Cyclin B (upper panel) and CDC2 (middle panel) levels were detected after SM or SPC treatment by western blot using 150 oocytes in each sample. The results are shown along with those of control oocytes cultured without any drugs. Samples were collected from culture medium with or without SM or SPC at 0 h, 24 h and 30 h.

To detect whether a GPR3 ligand, sphingosylphosphorylcholine (SPC), affects meiotic maturation, porcine oocytes were cultured in the presence or absence of 1 to 10 µM SPC. Negative control was sphingomyelin (SM) (10 µM), a phospholipid (a non-ligand for GPRs) present in the plasma membrane. Oocytes cultured in the presence of 10 µM SPC showed a significant delay in the GVBD (Fig. 6A). However, oocytes cultured in SM showed no difference in GVBD rate at 24 h as compared with the control. In porcine oocytes cultured in the presence of 10 µM SPC, the GVBD rate was only 54.23% at 24 h, whereas 82.96% control oocytes underwent GVBD. Even when cultured for 30 h, the GVBD rate still remained with the same tendency in the three groups: GVBD rates were 60.89% (SPC), 88.38% (SM), and 84.86% (control), respectively (Fig. 6B).

Then we analyzed cyclin B and CDC2 levels in porcine oocytes with SM or SPC treatment. As shown in Fig. 6C, cyclin B was undetected in porcine oocytes at the GV stage, but dramatically increased after culture for 24 h and 30 h in normal maturation medium without any ligands, while the CDC2 level remained almost unchanged. When porcine oocytes were cultured in medium containing SM (Fig. 6C), the similar expression of Cyclin B and CDC2 were detected. However, only faint bands of cyclin B were detected until 24 h and 30 h of culture in SPC treatment group, respectively. The CDC2 level of oocytes with SPC treatment remained relatively stable.

## Discussion

Studies have demonstrated that constitutively activated G protein-coupled receptor 3 (GPR3) is one of the critical molecules responsible for maintaining meiotic arrest in mouse and *Xenopus* oocytes [23]. Previous reports also indicated that GPR3 was localized primarily in the mouse oocyte itself and the constitutive activity of GPR3 in the oocytes is sufficient to produce the amount of cAMP required to maintain meiotic arrest [7]. Here, we investigated the localization and expression of GPR3 in porcine oocytes, and provided evidence for its important role in regulating meiotic resumption.

GPR3 belongs to the family of GPCRs with seven transmembrane domain motifs, and it is involved in the regulation of cAMP through a Gs protein in oocytes [1]. Although previous studies revealed that GPR3 was mainly expressed in the oocyte itself, there is no report showing its exact localization in oocytes. It was indicated that native levels of G-protein-coupled receptors are generally difficult to detect using antibodies because the level of these proteins in cells are low [3], [16], [27], thus it was difficult to detect specific labeling of GPR3 in mouse ovary using commercially available antibodies as reported by other groups [3], [26]. In our present study, the localization of GPR3 was first detected by immunofluorescence, and the results showed that GPR3 was indeed concentrated at the plasma membrane at all stages of porcine oocyte maturation, which was consistent with its characteristics of seven transmembrane domain motifs, receiving signals from the follicular environment to maintain meiotic arrest. However, it is worth noting that in the GV stage, a very clear positive signal was detected at the nuclear membrane, which strongly indicated that GPR3 may be directly involved in GVBD. Western blot results also indicated that GPR3 expression displayed a maximal level at the GV stage. After GVBD, the concentration of GPR3 in oocytes was decreased and remained unchanged thereafter. In previous studies, evidence showed that activated GPR3 maintained meiotic arrest through the Gs/cAMP signaling pathway [23], [28]. It was also proved that GPR3 and PDE3A are primary determinants of cAMP levels necessary to maintain meiotic arrest in mouse oocytes and that other mechanisms contributing to cAMP levels are not sufficient to maintain meiotic arrest [29]. Our results also indicate that GPR3 is involved in maintaining cAMP levels and regulating meiotic arrest in porcine oocytes.

Injection of Myc_6_-GPR3 mRNA or GPR3 siRNA into the cytoplasm was performed to examine its effect on meiotic arrest. It is well established that meiotic arrest is regulated by cAMP levels within the oocytes [9], [30]. Since the degradation of cAMP during meiotic resumption in mouse oocytes is achieved by phosphodiesterase (PDE3) [12], [31], spontaneous maturation of oocytes isolated from their follicles can be prevented by cAMP phosphodiesterase inhibitors, such as HX or IBMX, which are often added to prevent spontaneous cAMP degradation [9], [12], [23]. The presence of PDE3 in porcine oocytes had been reported, and previous research also observed the delay of GVBD after IBMX treatment in porcine oocytes [14], [15]. Moreover, cAMP levels decrease in oocytes following removal from follicles [32], as well as in isolated oocytes after removal of IBMX [4], [33]. Thus, we determined the effect of GPR3 in oocytes cultured in medium with or without HX.

First, Myc_6_-GPR3 was injected. When oocytes injected with Myc_6_-GPR3 mRNA were cultured in medium without HX, GVBD was significantly inhibited. The failure of GVBD might be attributed to the high cAMP and cGMP concentrations. The presumption was indeed confirmed by cAMP level and cGMP level detection. After Myc_6_-GPR3 mRNA injection, cAMP and cGMP concentrations were still maintained at a high level, suggesting that the overexpression of GPR3 in porcine oocytes elevated the activity of Gs protein and maintained high cGMP and cAMP levels. Our findings strongly suggest that GPR3 plays an important role in maintaining meiotic arrest in porcine oocytes.

Next we injected porcine oocytes with GPR3 siRNA followed by culture in medium containing HX. In the presence of HX, we found a significant promotion of GVBD by GPR3 siRNA injection, while most of the porcine oocytes without GPR3 siRNA injection remained at the GV stage. Previous reports also provided evidence *in vivo* and *in vitro* that inactivation of Gs or downregulation of GPR3 caused meiotic resumption in *pde3a* null mouse oocytes [10], [34]. GPR3 inactivation also caused a clear phenotype of leaky meiotic arrest [7]. We also determined the cAMP and cGMP levels of porcine oocytes injected with GPR3 siRNA in the presence of HX; as a result, a significant decrease in cAMP or cGMP levels was observed. Our findings suggest an important function for the GPR3-Gs-dependent synthesis of cAMP and cGMP in regulating meiotic resumption within porcine oocytes. It had been established that cGMP inhibited hydrolysis of cAMP by the phosphidiesterase PDE3A [35]. The inhibition maintained a high level of intra-oocyte cAMP and thus blocked meiotic progression.

To further analyze the effect of GPR3 on porcine oocyte meiotic arrest, we also determined the activity of MPF after injection of Myc-6-GRP3 mRNA or GRP3 siRNA. It had been reported that MPF is a critical meiotic regulator, which can be inhibited by high cAMP levels and subsequent high PKA activity through many signaling pathways regulating its activation [12], [36], [37]. It was further reported that cyclin B synthesis began at approximately 18 h of culture of porcine oocytes isolated from ovarian follicles, and MPF activity was elevated significantly at 24 h of culture [38], [39]. In our present study, two MPF subunits, Cyclin B and CDC2 levels were detected after culture in maturation medium. We found significant elevation of cyclin B levels in oocytes injected with GPR3 RNAi at 30 h of culture with HX, and inhibition of cyclin B accumulation in oocytes injected with Myc_6_-GPR3 mRNA. But the CDC2 level was almost unchanged. Previous studies reported that cAMP-PKA activity directly targets Cdc25B in prophase oocytes; Cdc25B phosphorylation results in its inhibition and thus inhibits MPF in mouse oocytes [17], [39], [40]. The cyclin B accumulation results were consistent with the GVBD data mentioned above.

The GPR3 ligand was also used to further confirm the effect of GPR3 on porcine oocytes. GPR3 is a subfamily of GPCR receptors that recognize SPC as ligands. SPC belongs to a class of lipid signaling molecules affecting target cells through activation of the cell surface's seven transmembrane receptors or as intracellular second messengers [41], [42]. In frog oocytes, meiotic resumption was caused by ceramide [43] and lipid synthesis was affected by progesterone [44]. Studies have suggested that lipid metabolites may play a role during meiotic maturation in frogs [44] and mouse [1]. When mouse oocytes were removed from the follicular environment, the decrease in ligand concentration may decrease GPR3 activity, allowing spontaneous maturation [1]. Our result showed that the GPR3 ligand did not alter the distribution of GPR3, but inhibited/delayed spontaneous oocyte maturation in porcine oocytes, which indicated that ligand through the way of regulating the GPR3 activity and further influence the expression of CyclinB, providing further evidence in support of the important role of GRP3 in oocyte meiotic arrest.

Taken together, we have shown for the first time the localization and expression of GPR3 in porcine oocytes. Reduction of GPR3 specifically in oocytes caused meiotic resumption when cultured in HX-containing medium. Overexpression of GPR3 inhibited meiotic resumption. These effects were mediated by the GPR3-cAMP/cGMP signaling pathway and MPF activity.

## Materials and Methods

### Chemicals and antibodies

All chemicals used in this study were purchased from Sigma Chemical Company (St. Louis, MO), unless otherwise noted. The cAMP complete Elisa kit and cGMP complete Elisa kit were purchased from Enzo Life Science (Enzo, NY). GPR3 antibody and Cyclin B antibody were purchased from Abcam (Abcam, NY). CDC2 antibody was purchased from Santa Cruz Biotechnology, Inc (Santa Cruz, CA). SPC and SM were also purchased from Abcam (Abcam, NY).

### Collection and preparation of porcine oocytes

Porcine ovaries were obtained from prepubertal gilts at a local slaughterhouse and transported to the laboratory within 1 hour in a thermos bottle. The ovaries were maintained in 0.9% NaCl solution containing penicillin G (75 mg/mL) and streptomycin sulphate (50 mg/mL) at 34°C∼36°C for the entire time of the transport. Cumulus-oocytes complexes (COCs) were aspirated from antral follicles (3–6 mm in diameter) of ovaries with an 18-gauge needle fixed to a 20 ml disposable syringe.

After three rinses in washing medium (TCM-199 medium supplemented with 2.2% NaHCO_3_), COCs with uniform cytoplasm and at least four layers of compact cumulus cells were selected for culture. To obtain denuded oocytes (DOs) for injection, COCs were mechanically denuded using a vortex instrument to remove all cumulus cells surrounding the oocytes in 0.5 ml TCM-199 containing 300 IU/ml hyaluronidase.

### Culture of porcine oocytes

The culture of COCs and DOs was performed according to the methods used in our previous studies [45], [46], [47]. The basic medium was TCM-199, supplemented with 0.1% PVA (w/v), 0.91 mM sodium pyruvate, 75 µg/ml potassium penicillin G and 50 µg/ml streptomycin sulphate. Two kinds of maturation culture models were used in our study: 1) normal maturation model: oocytes were cultured in basic medium with 0.57 mM cysteine, 0.5 µg/ml FSH, 0.5 µg/ml LH, and 10 ng/ml EGF, and 2) inhibition-induction maturation model: the oocytes were cultured in basic medium supplemented with 4 mmol/L HX and 0.5 µg/ml FSH, to mimic intact follicles. Groups of 40 to 50 COCs or DOs were cultured in a 200 µl drop of maturation medium in 4-well dishes, covered by liquid paraffin oil for up to 44 h at 39°C in an atmosphere of 5% CO_2_ in air and saturated humidity.

### GPR3 plasmid construction

Total RNA was extracted from 100 porcine GV oocytes using RNeasy micro purification kit (Qiagen); cDNA synthesis kit (Takara) was used to generate the first strand cDNA, using poly (dT) as its primers. The following two primers were used to clone the full length of GPR3 cDNA by PCR. F: CCC CGG CCG GCC GAT GAT GTG GGG TGC AGG CA; R: CCC CGG CGC GCC CTA GAC GTC ACT GGG AGA ACG G. Next, PCR product was extracted with gel extraction kit (Promega), GPR3 cDNA was subcloned to pMD18-T vector (Takara) and transfected into TOP10 *E.coli* competent cells. Then we extracted plasmid (Tiangen), and the correct sequence was acquired by double enzyme cut (*Fse*I and *Asc*I). To detect the overexpressed protein, GPR3 cDNA was tagged with NH_2_-terminally-Myc_6_. For *in vitro* transcription reactions, the Myc_6_-GPR3 cDNA was subcloned into pCS2^+^.

### RNA synthesis

The Myc_6_-GPR3-pCS2^+^ plasmid was linearized by *Not*I and purified by the gel extraction kit (Qiagen). Capped mRNA was produced by the T3 message machine (Ambion), which was purified by using the RNeasy cleanup kit (Qiagen). The concentration of GPR3 mRNA was detected with a Beckman DU 530 Analyzer, and then diluted to high concentration (2.5 mg/ml) for overexpression or low concentration (0.4 mg/ml) for localization experiments.

### Microinjection of Myc6-GPR3 mRNA or GPR3 siRNAs

GPR3 mRNA microinjection was performed using a Nikon Diaphot ECLIPSE TE300 (Nikon UK Ltd., Kingston upon Thames, Surrey, UK) inverted microscope equipped with Narishige MM0-202N hydraulic three-dimensional micromanipulators (Narishige Inc., Sea Cliff, NY). 0.4 mg/ml or 2.5 mg/ml Myc_6_-GPR3 mRNA solution was injected into cytoplasm of the oocytes for localization or overexpression. The same amount of Myc_6_ mRNA was injected as control. Each experiment consisted of three separate replicates and approximately 150 oocytes were injected in each group. The ratio of GVBD was determined at the appropriate time points by counting after orcein staining using an inverted optical microscope.

For depleting GPR3, small interfering RNAs (siRNAs) of GPR3 (Ambion) was microinjected into the cytoplasm of porcine oocytes. 25 µM GPR3 siRNA was used: UGCCCUCACCUACUACUCAUU. The same amount of negative control siRNA was injected as control. After microinjection, the GV oocytes were cultured for 24 h or 30 h in TCM-199 medium with or without 4 mM HX. The ratio of GVBD was determined at the appropriate time points by counting after orcein staining using an inverted optical microscope.

### Nuclear status examination

The orcein staining method was used for examining the nuclear status of porcine oocytes, which was carried out as described previously [47]. Briefly, denuded oocytes were mounted on glass slides, and fixed in acetic acid/ethanol (1∶3, v/v) for at least 48 hours at room temperature (25°C). Then oocytes were stained with 1% (w/v) orcein in 45% (v/v) acetic acid, and examined under a light microscope at a magnification of ×100. Oocytes displaying clear nuclear envelopes which enclose filamentous or condensed chromatin were classified as germinal vesicle (GV) stage oocytes, and those not displaying nuclear membranes were classified as germinal vesicle breakdown (GVBD) stage oocytes.

### Immunoblotting analysis

Porcine oocytes at appropriate stages and oocytes injected with Myc_6_-GPR3 mRNA, GPR3 siRNA, control mRNA or control siRNA were collected in sodium dodecylsulfate (SDS) sample buffer and boiled for 5 minutes. Oocytes cultured with ligand or not were also collected for SDS sample. Immunoblotting was performed as described previously [48]. Briefly, the proteins were separated by sodium dodecylsulfate polyacrylamide gel electrophoresis (SDS-PAGE) and then electrically transferred to polyvinylidene fluoride membranes. Following transfer, the membranes were blocked in Tris-buffered saline Tween-20 (TBST, TBS containing 0.1% Tween 20) containing 5% skimmed milk for 2 hours, followed by incubation overnight at 4°C with anti-GPR3 (Abcam, ab55136), anti-myc (Invitrogen, P/N46-0603), anti-cyclin B (Abcam, ab72), and anti-CDC2 (Santa Cruz, sc-54) with dilutions of 1∶500, 1∶1000, 1∶500 and 1∶500, respectively. After washing in TBST 3 times, the membranes were incubated for 1 hour at 37°C with 1∶1000 horseradish peroxidase (HRP)-conjugated IgG. For detection of β-actin, the membrane was washed in the washing buffer (100 mM β-mercaptoethanol, 20% SDS, and 62.5 mM Tris, pH 6.7) for 30 minutes at 37°C. Then β-actin was assayed on the same membrane by using anti-β-actin antibody (1∶1000) and HRP-conjugated IgG. Finally, the membrane was processed using the SuperSignal West Femto maximum sensitivity substrate (Thermo Scientific). Each treatment was repeated at least three times.

### Immunofluorescent microscopy

Immunofluorescence was performed as described previously [47], [48]. For staining of GPR3, porcine oocytes were fixed in 4% paraformaldehyde in PBS for at least 30 minutes at room temperature. After being permeablized in PBS containing 1% Triton X-100 overnight at 37°C, they were blocked in PBS containing 1% BSA (blocking solution) for 1 hour at room temperature. Then oocytes were incubated overnight at 4°C with 1∶100 anti-GPR3 (Abcam) or 1∶100 anti-myc-FITC (Invitrogen, P/N46-0307), and oocytes without antibody incubation were used as control. After three washes in PBS containing 0.1% Tween 20 and 0.01% TritonX-100 (washing solution) for 5 minutes each, the porcine oocytes were labeled with 1∶100 FITC-conjugated IgG for 1 hour at room temperature, and then rinsed in washing solution 3 times. Nuclear status was determined after staining with Hoechst 33342 (10 µg/ml in PBS) for 10 minutes, and oocytes were then washed extensively. Finally, oocytes were mounted on glass slides and examined with a confocal laser scanning microscope (Zeiss LSM710 META, Germany). Each treatment was repeated at least three times.

### cAMP measurement

We determined cAMP by cAMP Complete Elisa Kit (Enzo Life Science) [49]. We lysed 20 porcine oocytes in 0.1 M HCl for 10 minutes at room temperature (with occasional vortexing), and then inspected the cells under a microscope to ensure uniform lysis. After centrifuging at 15,000 rpm for 15 minutes at 4°C to pellet the cellular debris, the supernatant was transferred to a new tube and stored at −20°C until assayed with the cAMP Complete Elisa Kit. cAMP levels were measured according to the kit's provided protocol and manufacturer's instructions.

### cGMP measurement

We used cGMP Complete Elisa Kit (Enzo Life Science) to determine cGMP levels [49]. After different treatments, 20 porcine oocytes were lysed in 0.1 M HCl for 10 minutes at room temperature; to ensure uniform lysis, the samples were further incubated for an additional 10 minutes. Then, porcine oocytes were centrifuged at 15,000 rpm for 15 minutes at 4°C, and the supernatant was transferred to a new tube and stored at −20°C until assayed with the cGMP Complete Elisa Kit. cGMP levels were detected according to the kit's provided protocol and manufacturer's instructions.

### Drug treatments of oocytes

Sphingomyelin (SM) (Abcam) was prepared as liposomes, according to the manufacturer's instructions. Sphingosylphosphorylcholine (SPC) (Abcam) was dissolved in water [1]. Porcine oocytes were collected in TCM-199 medium with or without 1 or 10 µM SPC or 10 µM SM and placed in 200 µl drops of the same medium under mineral oil. The oocytes were scored for meiotic maturation at 0 h, 12 h, 24 h, 30 h and 36 h, respectively. Progression of porcine oocyte meiotic maturation was scored by monitoring the breakdown of germinal vesicles (GVBD) after staining with orcein. Oocytes showing clear nuclear membranes (germinal vesicle, GV) and nucleoli were classified as GV stages, while those without visible nuclear structure were classified as GVBD stages. Oocytes treated by SPC were collected at various stages to detect the distribution of GPR3 and the expression of cyclin B and CDC2.

### Statistical Analysis

GVBD percentages from three repeated experiments were expressed as mean ± SEM. For cAMP and cGMP concentration, values are expressed as pmol/oocyte ± SEM. All frequencies were subjected to arcsine transformation. The transformed data were statistically compared by ANOVA using SPSS version 10.0 software (SPSS Inc., Chicago, IL) followed by Student-Newman-Keuls test. Different values were considered significant when the P value was less than 0.05.

## Supporting Information

Figure S1 **Scanned oocytes in consecutive planes.** Oocytes in GV stage were scanned in numerous planes to see entire oocytes volume. Green, GPR3; Blue, DNA (chromosomes). Bar = 10 µm.
(TIF)
