# Supplementary figures and images for: The G Protein Coupled Receptor 3 Is Involved in cAMP and cGMP Signaling and Maintenance of Meiotic Arrest in Porcine Oocytes
Source: PLoS One. 2012 Jun 7;7(6):e38807. doi: 10.1371/journal.pone.0038807 (PMC3369857; doi:10.1371/journal.pone.0038807)

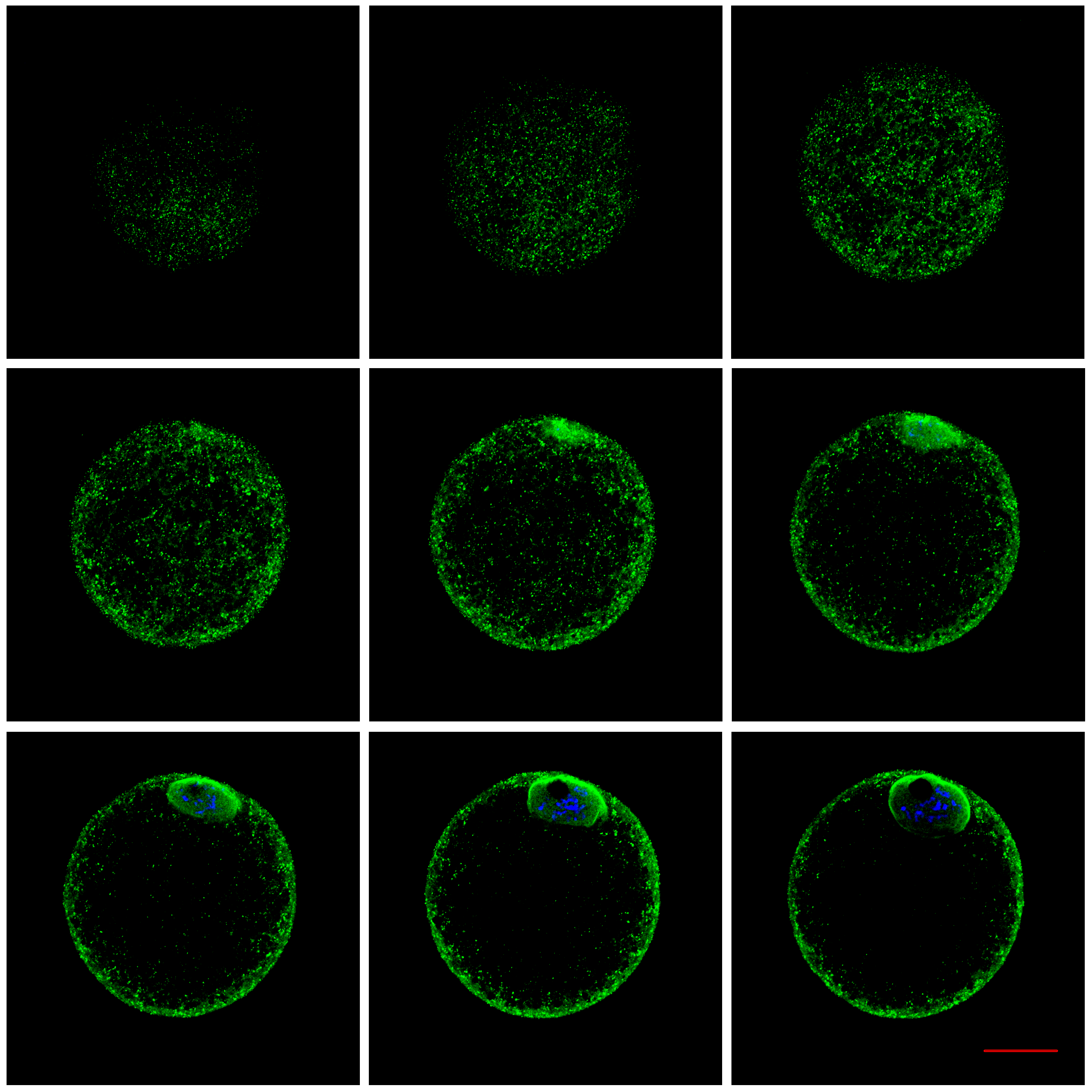

Supplement: Figure S1 — Scanned oocytes in consecutive planes. Oocytes in GV stage were scanned in numerous planes to see entire oocytes volume. Green, GPR3; Blue, DNA (chromosomes). Bar = 10 µm. (TIF) [file pone.0038807.s001.tif]
